# The Timing of the Maternal Recognition of Pregnancy Is Specific to Individual Mares

**DOI:** 10.3390/ani13101718

**Published:** 2023-05-22

**Authors:** John R. Newcombe, Juan Cuervo-Arango, Sandra Wilsher

**Affiliations:** 1 Warren House Farm, Equine Fertility Clinic, Brownhills WS8 6LU, West Midlands, UK; john@warrenhousevets.com; 2 Departamento de Medicina y Cirugía Animal, Facultad de Veterinaria, Universidad Cardenal Herrera-CEU, CEU Universities, 46115 Alfara del Patriarca, Spain; juan.cuervo@uchceu.es; 3 Sharjah Equine Hospital, Sharjah 61313, United Arab Emirates; 4 The Paul Mellon Laboratory of Equine Reproduction, “Brunswick”, Newmarket CB8 9BJ, Suffolk, UK

**Keywords:** mare, maternal recognition of pregnancy, luteostasis, luteolysis, cycle length, embryonic vesicle diameter, embryo reduction

## Abstract

**Simple Summary:**

The mechanism which ensures a mare recognises the presence of a pregnancy and receives an embryonic signal to prevent her returning to oestrus is known as maternal recognition of pregnancy (MRP) and occurs approximately 12 days after ovulation. We do not fully understand the MRP signal in the mare or the mechanisms involved in how it prevents lysis of the corpus luteum, the progesterone secreting structure on the maternal ovaries. This experiment aimed to determine if the timing of the MRP signal was specific to individual mares by examining when luteostasis (maintenance) of the corpus luteum reliably occurred in individuals following embryo reduction. Singleton (n = 150) and synchronous twin pregnancies (n = 9) were reduced in 10 individuals (5–29 reductions/mare) at pre-determined time points within days 10–14 of pregnancy. Individual mares showed a significant variation in when they consistently entered a period of luteostasis following embryo reduction (272–344 h post-ovulation). The presence of twins, the size of the embryonic vesicle, the interovulatory period and mare age did not factor into the timing of MRP in the individuals. The factors and mechanisms underlying the individuality in the timing of MRP were not determined and warrant further study.

**Abstract:**

The present experiment aimed at determining whether the timing of the maternal recognition of pregnancy (MRP) was specific to individual mares by determining when luteostasis, a failure to return to oestrus, reliably occurred in individuals following embryo reduction. Singleton (n = 150) and synchronous twin pregnancies (n = 9) were reduced in 10 individuals (5–29 reductions/mare) at pre-determined time points within days 10 (n = 20), 11 (n = 65), 12 (n = 47), 13 (n = 12) or 14 (n = 15) of pregnancy. Prior to embryo reduction, the vesicle diameter was measured in 71% (106/150) of the singleton pregnancies. The interovulatory interval (IOI) was recorded on 78 occasions in seven of the mares in either non-pregnant cycles (n = 37) or those in which luteolysis followed embryo reduction (n = 41). The earliest time post-ovulation at which the embryo reduction resulted in luteostasis in an individual was 252 h (mid-Day 10). Consistency in luteostasis following embryo reduction showed individual variation between mares (272–344 h). Binary logistic regression analysis showed an individual mare effect (*p* < 0.001) and an effect of the interval post-ovulation at which embryo reduction was undertaken (*p* < 0.001). However, there was no significant effect of vesicle diameter at the time of embryo reduction (*p* = 0.099), nor a singleton or twin pregnancy (*p* = 0.993), on the dependent of luteolysis or luteostasis. The median IOI between individual mares varied significantly (*p* < 0.05) but was not correlated to the timing of MRP. The timing of MRP varied between the mares but was repeatable in each individual. The factors and mechanisms underlying the individuality in the timing of MRP were not determined and warrant further study.

## 1. Introduction

Early pregnancy is established and then maintained by elevated blood progesterone concentrations resulting from continuing progesterone secretion by the primary corpus luteum. For this to occur beyond the time of endogenous prostaglandin release from the endometrium, the maternal system must recognize the presence of an embryo, otherwise progesterone levels will fall to basal, with a consequent return to oestrus and expulsion of the conceptus. The mechanism which ensures the mare recognizes the presence of a pregnancy and receives an embryonic signal which prevents the lysis of the corpus luteum, is known as maternal recognition of pregnancy (MRP) [1]. Although the MRP signal is known in large domestic animal species, such as the cow, pig and sheep [2], it remains to be elucidated in mares.

In the non-pregnant cyclic mare, progesterone concentrations begin to fall approximately 14 days after ovulation, which is coincidental with maximal levels of prostaglandin (PGF2α), measured by the presence of its metabolite (PGFM) in the uterine vein, uterine lumen and endometrium [3,4,5,6,7,8,9]. In addition, the enzyme responsible for the release of arachidonic acid, and hence the initiator of the release of PGF2α, is highest in non-pregnant mares at day 14 [10]. In contrast, PGF2α release from the endometrium is attenuated in pregnant mares during the time of expected luteolysis [6,7].

Oxytocin also plays a role in the luteolytic cascade, with the binding of endometrial oxytocin to its receptor (OXTR) in the endometrium believed to cause the pulsatile release of PGF2α [11,12]. Both endometrial and luteal oxytocin concentrations show changes based on the pregnancy status and the day post-ovulation [13], while around the timing of MRP the transcriptional levels of OXTR are similar between pregnant and non-pregnant mares [14,15]. However, OXTR protein levels decrease in pregnant animals [11,12,13], suggesting that OXTR are modulated at the post-transcription level rather than at the transcriptional one. In cycling mares, OXTR concentrations increase approximately threefold on day 14 compared to days 10 and 18, with no such increase seen in pregnant animals [12], suggesting that suppression of the expression of OXTR needs to be initiated before day 14 in pregnant mares.

Although the equine endometrium produces oxytocin [16,17,18], until recently the consensus of opinion has been that the equine CL does not do likewise [19,20]. Hence, in equids, unlike ruminants [21], CL-derived oxytocin was not thought to stimulate the luteolytic pulses of endometrial PGF2α to lyse the CL at the end of dioestrus. However, recent work by Diel de Amorin [18] localised oxytocin to the CL, although the role it may play in luteolysis or luteostasis has not been determined. 

However, regardless of the precise mechanism that prevents luteal regression and results in a functional corpus luteum, the MRP signal must be given before the release of PGF2α from the endometrium around day 14. This is evidenced by the fact that in a clinical setting, luteal failure is rarely seen following spontaneous embryo failure after day 14 or 15 of pregnancy, and exogenous PGF2α is needed to return the mare to oestrus [22,23]. Even though the MRP signal is delivered before day 14, the exact moment when the signal abrogates luteolysis is unclear. Although earlier literature suggested that the MRP signal occurred around day 10, more recent work suggests that it is later. For example, day 12 recipient mares can become pregnant following transfer of a day 10 embryo, whereas transfer at day 14 is unsuccessful, with the mares returning to oestrus [24]. Furthermore, the suppression of prostaglandin-endoperoxide synthase 2 (formerly COX-2), the enzyme involved in the conversion of arachidonic acid to prostaglandin H2, occurs as late as day 13 of pregnancy [25]. Hence, the suggested time frame for the liberation of the MRP signal is between days 12–14. However, a more precise timing has not been determined. Whether the signal is a one-off, short-lived event or a continuum of events, as suggested by Sharp et al. [26], is also unclear. However, Wilsher et al. [27] recently demonstrated that manual reduction of day 11 embryos 24 or 12 h after transfer to recipient mares on either day 10, 11, 12 or 13 post-ovulation resulted in 93% (25/27) entering a period of luteostasis. An extension of this work demonstrated that in vitro rupture of an in vivo recovered day 11 embryo and its subsequent transfer, along with the medium in which it was ruptured, resulted in significantly more day 12 recipients entering a period of luteostasis compared to day 11 recipients (75% vs. 12.5%; 6/8 vs. 1/8; *p* = 0.04, respectively) [28]. These studies suggest that the MRP signal can be given over a relatively short time period and would appear to confirm day 12 as the optimum time point to suppress PGF2α release from the endometrium.

The data from Wilsher et al. [27] and Newcombe et al. [29] also suggests that a single embryo reduction on or after day 12 results in the majority of mares entering a period of luteostasis. In these studies, it was noted that when individual mares underwent several reductions, there appeared to be consistency in the timing of when MRP was triggered. The present study aimed to extend this work and confirm whether the timing of the MRP signal was indeed specific to individual mares.

## 2. Materials and Methods

### 2.1. Mares

Ten mares of mixed breed and aged between 6–28 years at the end of the experiment were used for the study. The mares were kept outside, maintained on hay ad lib and supplemented with concentrates as required to maintain good bodily condition. The nature of the experiment meant that the mares were used on multiple occasions. 

### 2.2. Experimental Protocol

#### 2.2.1. Insemination and Timing of Ovulation

**Figure 1:**
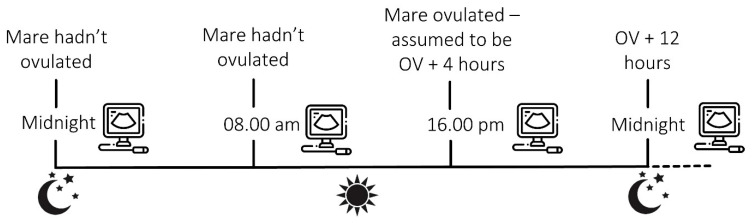
An example of calculation of the ovulation time. Ultrasound examinations of the reproductive tract were undertaken at 8-h intervals. In this example, a mare scanned at 08.00 h had not ovulated but had ovulated during the following examination at 16.00 h. As ovulation could have occurred at any time point in the preceding 8 h, ovulation was estimated as midway between the two examinations and the mare was assumed to be 4 h post-ovulation.

Over four breeding seasons and numerous cycles, ten individual mares were inseminated during oestrus when a follicle > 35 mm was present on an ovary in combination with uterine oedema. At the time of insemination, an ovulatory drug was administered to induce ovulation approximately 36–40 h later. Ultrasound examinations of the reproductive tract were undertaken every 8 (±1) h from late oestrus until the time of ovulation. As ovulation could have occurred at any time during the 8-h window between the ultrasound examinations, the time of ovulation was determined to be 4 h before it was first detected ultrasonographically (Figure 1). Ultrasound examinations for pregnancy began on day 9 or 10 and were repeated every 8 h thereafter until an embryo was first positively identified, until the time of embryo reduction or until non-pregnancy was confirmed.

#### 2.2.2. Embryo Reductions

A total of 150 singleton pregnancies were reduced in 10 individual mares at specific time points corresponding to eight or four hourly intervals within day 10 (n = 20), day 11 (n = 63), day 12 (n = 44), day 13 (n = 10) or day 14 (n = 13). Once an individual mare became luteostatic following an embryo reduction, more reductions were undertaken around this time point to confirm an individual’s response. Nine sets of synchronous twins were also reduced on day 11 (n = 2), day 12 (n = 3), day 13 (n = 2) and day 14 (n = 2). The data on whether these reductions resulted in luteostasis or luteolysis was excluded from the main data set and is discussed separately.

At a pre-determined time post-ovulation, embryos were reduced using compression and rupture, either with an ultrasound transducer or digitally between the finger and thumb. If twins were present, both were ruptured at the same time. Mares in which an embryo was reduced, and in which a subsequent examination revealed a second embryo presumed to be from an asynchronous ovulation, were excluded from the data. While larger embryos could sometimes be compressed and ruptured in situ, the majority had to be manipulated to the tip of a uterine horn or to the cervix, by compressing adjacent sections of uterine horn, where they could be isolated and manually reduced. Prior to embryo reduction, the diameter of the embryonic vesicle was measured in 70.6% (106/150) of singleton pregnancies using the caliper on the ultrasound machine. The mean of the two measurements was recorded.

#### 2.2.3. Monitoring Mares for Luteostasis or Luteolysis

Following reduction, the mares were re-examined for evidence of luteal regression and a return to oestrus. Continued luteostasis was determined as a functional CL with blood flow on colour doppler, a lack of endometrial oedema up to and including day 25 without a previous return to oestrus or any secondary ovulation(s). Mares which entered a period of luteostasis following embryo reduction were given an intramuscular injection of 250 µg of a prostaglandin analogue on or after day 25 to lyse the CL and return them to oestrus. A failure to maintain luteostasis following embryo reduction was determined as a return to oestrus from approximately day 17 with disappearance of the CL and the presence of endometrial oedema followed by ovulation. On 78 occasions in seven of the study mares, the cycle length in both the non-pregnant cycles (n = 37) and those in which the mares returned to oestrus following embryo reduction (n = 41) was recorded. Cycle length was defined as the number of days from ovulation (day 0) until the subsequent ovulation and was only recorded in mares that were followed to ovulation during oestrus and in cycles where there was no hormonal induction of ovulation.

#### 2.2.4. Statistical Analysis

All the statistics were performed using a computer software package (SigmaPlot vs. 11.0, SPSS Inc. Chicago, IL, USA). Binary logistic regression was undertaken with luteolysis or luteostasis following embryo reduction as the dependent variable and mare ID, mare age, time in hours post-ovulation when the reduction was undertaken, embryonic vesicle diameter at the time of the reduction of singletons when recorded, and the presence of a single or twin pregnancy reduction as the independent variables. Significance was set at *p* < 0.05.

The data for embryonic vesicle diameters was non-parametric, hence median (25–75% range) values were reported. Further analysis of the data to determine whether there was an effect of embryo vesicle diameter on mares becoming luteostatic or luteolytic after embryo reduction on set days or intervals was undertaken using a Kruskal–Wallis one way ANOVA on ranks with a post-hoc Dunn’s test, if required. Significance for all the tests was set at *p* ≤ 0.05. The data for cycle length was reported as the mean (±SEM). A two-way ANOVA with a post-hoc Holm–Sidak comparison was used to determine if cycle status (luteolytic cycle following embryo reduction or non-pregnant cycle) and mare ID influenced the cycle length.

## 3. Results

### 3.1. The Effect of the Embryo Reduction on Luteostasis

The number of reductions in singleton pregnancies, which resulted in either luteolysis or luteostasis at each time point for each of the 10 mares, is shown in Table 1. When considering only the singleton embryo reductions undertaken on a specific day, luteostasis occurred on day 10 in 10% (2/20), day 11 in 52.4% (33/63), day 12 in 63.6% (28/44), day 13 in 60% (6/10) and on day 14 in 92.3% (12/13) of the mares. Significantly more mares entered a period of luteostasis following embryo reduction on days 11, 12, 13 or 14 versus day 10 (*p* ≤ 0.013 in all cases), and significantly more mares were luteostatic post-embryo-reduction at day 14 versus day 11 (*p* = 0.019). As would be expected, regression analysis showed that the interval post-ovulation when reduction was undertaken was a significant factor for a mare becoming luteostatic or luteolytic post-reduction (*p* < 0.001).

The earliest time point in which embryo reduction of a singleton pregnancy resulted in luteostasis varied between the ten individuals from between 252 and 332 h. Hence, this variation was from as early as mid-day 10 to late day 13 post-ovulation. However, luteostasis at the earliest time point was not always consistently achieved following reduction, and some mares showed an inconsistent response for a short period of time, in which only a proportion of the reductions achieved luteostasis, before which consistent luteolysis occurred and after which luteostasis occurred. Inconsistency in the response did not span more than 24 h in any mare (Table 1). Consistency in achieving luteostasis with every reduction and all those thereafter varied between 272 and 344 h, a period of 72 h (Table 1). Binary logistic regression analysis showed a strong individual mare effect (*p* < 0.001) on the dependent of luteolysis or luteostasis.

### 3.2. Embryo Recognition and Vesicle Size

Embryonic vesicles were first viewed ultrasonographically between 220–268 h (days 9–11) post-ovulation when they were 1.5–3 mm in diameter. The majority of embryos were first seen between 228 or 236 h (late day 9), although some small-for-age embryos were not identified until later. Substantial pressure was needed to rupture the smaller vesicles, which were moved to the cranial tips of the horns or to the caudal body prior to reduction. It was also noted that regardless of their diameter, some vesicles ruptured significantly more easily than others.

Binary logistic regression model showed that embryo vesicle diameter at the time of reduction was not a significant factor in mares becoming luteostatic or luteolytic following reduction (*p* = 0.099). Larger vesicle diameters were, by the nature of embryo development, more likely to be recorded at later time intervals post-ovulation when luteostasis was, in general, more likely to occur.

The median (25–75% range) embryonic vesicle diameter (excluding twin vesicles) prior to reduction on days 10–14 for mares that became luteostatic versus those which returned to oestrus (luteolytic) are detailed in Table 2. For each day, no significant difference existed in the size of the vesicle in those mares that did or did not become luteostatic. Even when the time points within days 11 and 12 were examined, the size of the embryonic vesicle prior to rupture did not affect the likelihood of a mare entering a period of luteostasis (Table 3). For the three mares which had ≥ four reductions resulting in luteostasis and ≥four reductions in luteolysis on an individual day, the median embryonic vesicle diameter was not significantly larger or smaller in those entering a period of luteostasis versus luteolysis following embryo reduction (Table 4).

To examine whether the mare (CSO) which had the earliest suppression of the luteolytic pathway (Table 1), and hence became luteostatic following embryo reduction at the earliest time point, had larger embryos potentially secreting more MRP signal, embryonic size was compared between this mare and two others (AA and PY, which had the 2nd and 3rd latest suppression points) on day 11 and 12. On neither day did mare CSO have significantly larger embryos than the other two mares.’ Indeed, this mare had the smallest median embryo diameter on both days compared to the other two mares.

### 3.3. Twin Embryo Reduction

A total of nine sets of twins were reduced in three of the mares in the study (ZA [n= five sets), AA [n = one set] and PY [n = three sets]). One set of twins in mare ZA was reduced at 308 h (Day 12) and resulted in luteostasis, although in mare ZA, no singleton reduction caused luteostasis until 24 h later at 332 h (day 13; Table 1). However, two sets of twins in mare ZA reduced at 332 and 336 h (day 13) failed to cause a prolongation of the corpus luteum whereas two of the three singleton reductions did. Two further sets reduced in mare ZA at 340 h (Day 14) resulted in luteostasis in one case and luteolysis in the other, with the same result in two singletons reduced at the same time point. In the other mares (AA and PY), all twin reductions resulted in luteostasis, as did all but one of the singletons reduced at equivalent time points. The presence of a singleton or twin at the time of embryo reduction did not affect the likelihood of luteostasis or luteolysis in the binary logistic regression analysis (*p* = 0.993).

### 3.4. Cycle Length and Mare Age

Cycle length in seven of the ten individual mares used in the study, for which data was available, did not vary significantly between non-pregnant cycles and those in which a reduction was followed by luteostasis (Table 5). Indeed, there was relative consistency in the cycle length in individual mares. When considering all cycles, there were significant differences in the cycle length between individual animals (Table 5). The mare (AA) with the latest response to embryo reduction, in terms of becoming luteostatic, had the shortest cycle length. However, her cycle length was not significantly different in length to that of the mare (CSO) which entered a period of luteostasis at the earliest timepoints following embryo reduction (Table 1 and Table 5).

The binary logistic regression analysis showed no influence on the age of the mare on the likelihood of her becoming luteolytic or luteostatic following embryo reduction (*p* = 0.274).

## 4. Discussion

The time when the maternal recognition of pregnancy signal (MRP) occurs in the horse has been defined as occurring around day 12 post-ovulation (reviewed by [30]). In trying to pinpoint more precisely when this occurred, previous work by the authors inadvertently discovered that the time of delivery of the MRP signal appeared to be variable but repeatable in individual mares [27,30]. The present study expanded on that observation and determined that the timing of the MRP signal varied by up to 72 h between individual animals, as evidenced by the observation that embryo reductions before a certain time point post-ovulation result in luteolysis, whereas reductions after result in luteostasis. Furthermore, the time of delivery of the MRP signal occurs with repeatability in individual mares within a post-ovulation window of ≤24 h for each animal.

Although the window of embryo reduction resulting in consistent luteostasis in all 10 mares varied between 272 and 344 h (11 days 4 h–14 days 4 h), the MRP signal must have been liberated for some undeterminable period prior to this time point. Alternatively, crushing the embryonic vesicle and the continuing presence of the remains of the capsule, trophoblast or blastocoele fluid may have caused some degree of persistence of the MRP signal. Both possibilities could, of course, be at play. In regard to the latter theory, a previous study demonstrated that the transfer of ruptured day 11 embryos (trophoblast, capsule and media containing the released blastocoele fluid) into day 10, 11, 12 or 13 recipient mares resulted in 0% (0/8), 30% (3/10), 73.3% (11/15) and 20% (2/10) of the mares, respectively, entering a period of luteostasis compared to 0% (0/10), 20% (2/10), 22.2% (4/18) and 0% (0/6) of the control recipient mares receiving only transfer media at the same stages. A significant difference existed between the control and treated animals on day 12 [28], unpublished data S Wilsher. These observations suggest that there was an influence on the luteolytic pathway for some time after the reduction of an embryo in the uterus, but this was short-lived and only effective around day 12 in most mares. So, even allowing for an extended effect of the MRP signal for a limited period following embryo reduction in the present experiment, there was still significant variability between individual mares.

One might assume that either larger embryos or twin embryos, due to their increased surface area and blastocoele cavity for production/secretion of a luteostatic factor and their greater contact area with the endometrium, would be more capable of suppressing luteolysis at a given time point in an individual animal. However, this did not appear to be the case within the time period leading up to when all the reductions caused luteostasis, and in which some reductions did and some did not result in luteostasis, since it was not necessarily either the larger vesicles or twin pregnancies that caused prolongation of the CL. Clinically, it has been noted that many small for gestational age vesicles (SFGA) appear capable of giving the MRP signal and prolonging the lifespan of the CL, even though they fail at a later time point [23]. Whereas, other SFGA vesicles seen on days 12–15 do in some instances fail to block luteolysis at days 14–15 with a resulting return to oestrus [22]. In an embryo transfer model, day 10 embryos transferred to day 12 recipients were able to block luteolysis despite being, in effect, small for gestational age with respect to uterine age [31]. Furthermore, experimentally, Wilsher et al. [27] found no discernible difference in the size of day 11 embryos on their ability to cause luteostasis when transferred to day 10, 11, 12 or 13 recipient mares. However conversely, in a model of extreme embryo asynchrony, transferring day 10 embryos to day 3 recipient mares, embryo diameter was a factor in future survival to the heartbeat stage. Hence, in their ability to give the MRP signal, it was the smaller embryos at the time of transfer that had the greater likelihood of surviving [31]. The data from the present study would also suggest that around the time of MRP, the presence of an embryonic vesicle, rather than its size, was the determining factor in the effective transmission of the signal to prevent luteolysis. Hence, uterine stage (time from ovulation) rather than embryo age and size appeared to be the overriding influence in determining when MRP occurs.

In all biological systems, there will inevitably be differences between individuals of the same species, so it is no surprise that the timing of the MRP signal in the mare shows biological variation. Such variations in the mechanisms controlling the luteolytic cascade are controlled by genes, whose expression or otherwise is likely to be orchestrated by the reproductive hormones that control the oestrous cycle from one ovulation to the next. As cycle length is known to vary between individuals, this was also examined in the present study to determine whether it had an influence on the timing of when MRP occurs.

The average length of the oestrus cycle or interovulatory interval (IOI) was around 21 days, with a mean (and range of means) of 21.7 (19.1–23.7) for the whole oestrous cycle, 6.5 (4.5–8.9) days for oestrus and 14.9 (12.1–16.3) for dioestrus reported by Ginther [32] from a compilation of ≤26 references. Factors that have been ascribed to variation in the length of the oestrous cycle include season [32,33,34], mare status (e.g., lactating vs. non-lactating) [35], and breed (e.g., ponies vs. horses) [32]. Variations have been attributed predominantly to the length of oestrus rather than dioestrus [33,34]. The length of oestrus, in both induced and spontaneous cycles, has been shown to influence the chances of pregnancy in mated mares and those used as embryo transfer recipients [36,37,38]. In addition, a more recent study found a positive correlation between endometrial oedema during oestrus and progesterone levels on day 14 of a pregnancy [39]. In looking for possible reasons for improved fertility from a longer oestrous period, a further study examined 17 genes expressed in the endometrium in anoestrous mares following the administration of exogenous oestradiol to mimic a long or short oestrous period, with control mares receiving no treatment prior to progesterone administration. Four days later, endometrial biopsies showed alterations in fibroblast growth factor-2 (FGF-2) and uterocalin (P19) related to the length of the preceding oestrus [40]. Although Silva et al. [40] only examined genes believed to contribute to embryo development and viability, there is no reason to think that the expression of other endometrial genes involved in both the luteolytic cascade and embryo–maternal dialogue around the time of MRP and would not, likewise, be influenced by reproductive hormones.

However, in the present study, the interovulatory interval (IOI) in seven of the mares for oestrous cycles when either embryo reduction did not result in luteostasis, or in unmated cycles, failed to show a relationship between the length of the IOI and the timing of when embryo reduction consistently resulted in luteostasis. Indeed, the two mares with the earliest and latest post-ovulatory periods when MRP occurred following embryo reduction had the two shortest IOIs of the seven mares analyzed, although differences in the number of days of oestrus in each IOI was not recorded. As previously reported [32,41,42], repeatability in the length of the cycle in individual mares was observed.

In ruminants, the antiluteolytic effects of the MRP signal, interferon tau (IFNT), is based on the progesterone block theory [43]. In this model of cyclicity, progesterone (P4) acts to block the expression of receptors for oestrogen alpha (ESR1) and oxytocin (OXTR) for 10 days post-ovulation. Thereafter, P4 downregulates the expression of progesterone receptors (PGR) which, in turn, allows for the resurgence of ESR1 and OXTR in the endometrium. Pulsatile release of oxytocin (OXT) from the posterior pituitary gland and CL and its subsequent binding to the OXTR results in prostaglandin release from the endometrium on days 15–16, causing the demise of the CL and a return to oestrus. When an embryo is present, the secretion of IFNT silences the ESR1 gene, preventing oestradiol-induced expression of OXTR, and hence prevents OXT-induced luteolytic pulses of prostaglandin, thus preventing a return to oestrus (reviewed by [2]). In pigs, the series of events leading to MRP is different but in common with ruminants and all other mammals studied to date. Silencing the PGR in the uterine epithelia is a prerequisite for starting the processes that lead to the luteolytic cascade. A side issue to note here is that in an early pregnant animal PGR in the uterine stromal glands remain expressed and when progesterone binds to them, it triggers the production of progestamedins that aid conceptus development and the secretion of histotroph from endometrial glands [44]. In mares, the sequential and spatial expression of PGR in the endometrium of cyclic and early pregnant mares has been examined using various techniques [45,46,47,48,49,50] which demonstrated that both mRNA and localisation of PGR decline to a nadir in late dioestrus.

The pivotal role that progesterone plays in the luteolytic cascade in cows and ewes is illustrated by the shortening of the inter-oestrus period when exogenous progesterone is administered as a result of an advancement in the release of endometrial prostaglandin [51,52,53]. In one study, the effect on hastening luteolysis was shown to be dose dependent [53]. In horses, the luteolytic cascade is not as well understood, and the MRP signal is unknown, although components of the luteolytic cascade are gradually being unravelled reviewed in [30,54,55,56,57]. However, as with other mammals, it is presumed that progesterone plays a regulatory role in setting up the events that lead to the luteolytic cascade. Indeed, progesterone has been noted to be involved in the changes of endometrial gene expression around the time of MRP [14,58]. There is also some evidence in horses that low progestin levels post-ovulation alter the expression of PGR in the endometrium from a model impairing luteal function with cloprostenol treatment on days 0–3 of the cycle. So treated pregnant mares had delayed downregulation of PGR in the endometrial epithelium compared to control mares at day 14 [59]. Although unfortunately, neither peripheral progesterone serum concentrations nor the days of oestrus were investigated in the present study, it is possible that individual mares may have shown intrinsic differences in the rise or concentrations of progesterone post-ovulation, affecting gene expression involved in the luteolytic cascade/MRP. Conversely, the lack of any notable difference in the length of the oestrous cycle in the mare with the earliest set point for MRP versus the mare with the latest in the present study would appear to argue against this theory.

Age-related degenerative changes in the endometrium have also been shown to disrupt progesterone and oestrogen receptor expression, both elements of the luteolytic pathway [60]. However, despite the likelihood that older mares in the current study probably had a degree of endometrial fibrosis, no relationship between early and late transfer of the MRP signal and mare age could be determined.

One of the long-held tenets of early equine embryonic development is that embryo mobility around the uterus is required to deliver the MRP signal to the entire endometrial surface to suppress the release of PGF2α. Earlier studies have shown that the embryo migrates throughout the uterine body and horns in the period both before and after the time of MRP [61,62]. The level of activity (number of location changes per hour) increased from day 10–11, where the embryonic vesicle was more frequently seen in the uterine body, to peak on days 13–15 where it is seen increasingly in the uterine horns and especially at the base of the horns before its eventual fixation around days 16–17 in this latter location [61,62,63]. The results from the present study would suggest that the MRP signal was transmitted to the dam prior to the period of expected maximal activity, and well before embryonic mobility would normally cease at day 16–17. This calls into question whether the prime reason for the extended period of embryo mobility in the mare is transmission of the MRP signal.

## 5. Conclusions

In conclusion, the timing of when the MRP of signal can prevent luteolysis varies between mares. However, it shows repeatability in its timing in individual animals. The inherent set point for MRP in individuals is unrelated to the size of the vesicle at that time. In trying to elucidate the mechanisms underlying the MRP signal, mares at the extreme of the ‘MRP window’ may be useful investigative models. However, identifying such animals is time-consuming and requires numerous embryo reductions.

## Figures and Tables

**Table 1:** The number and timings of 150 singleton embryo reductions undertaken in 10 individual mares. The numbers within individual squares refer to the number of reductions that resulted in luteostasis over the total number of reductions at that time point. The timepoints when all reductions resulted in luteolysis are highlighted in orange. Those in which only a proportion of reductions resulted in luteostasis are highlighted in light green, and those in which all reductions resulted in luteostasis are highlighted in dark green.

Mare ID	Hours Post Ovulation When Embryo Reduction Was Undertaken																													No. of Singleton Reductions
	244	248	252	256	260	264	268	272	276	280	284	288	292	296	300	304	308	312	316	320	324	328	332	336	340	344	348	352	356	
	Day 10						Day 11						Day 12						Day 13						Day 14					
ZA			0/1		0/1		0/1		0/1		0/1		0/1		0/2		0/1		0/1		0/2		1/2	1/1	1/2	2/2	1/1	1/1	3/3	24
AA			0/1		0/1		0/2		0/1		0/2	0/1	1/2	2/3	2/5	1/2	2/2		1/1											23
GY	0/1		0/1		0/1		0/2		0/1		1/3		0/2	1/1	2/2	0/2	2/4	1/1											1/1	22
CO					0/1		0/1		0/1	0/1	2/2		1/1		1/1		1/1						1/1							10
DE									0/1	0/1	1/1	1/1	1/1																	5
IR							0/1		0/1		1/1		1/1								1/1		1/1							6
PS					0/1		0/1		1/1		1/1		1/1		1/1														1/1	7
PY					0/1	0/1	3/6	2/2	4/5		3/3	2/2	1/1			1/1													1/1	23
RS					0/1	0/1	1/2		2/2		2/3	0/1	1/1		1/1															12
CSO	0/1	0/1	1/2	0/1	1/2		2/3	1/1	1/1		2/2		2/2		1/1										1/1					18

**Table 2:** Median (25–75% range) embryonic vesicle diameter in 106 pregnancies (excluding twin pregnancies) prior to embryo reduction at set days post-ovulation. Within the days, no significant difference existed in the median embryo vesicle size between the luteolytic and luteostatic mares for the same day (Kruskal–Wallis one-way ANOVA on Ranks; *p* > 0.05).

Day of Embryo Reduction	Median (25–75% Range; n) Embryo Diameter (mm) of Vesicles at the Time of Reduction in Mares That Became:	
	Luteolytic	Luteostatic
Day 10	4.5 (4.2–5.4; n = 5)	6.6 (6.2–7.0; n = 2)
Day 11	7.1 (5.5–7.7; n = 21)	6.8 (5.1–8.3; n = 29)
Day 12	8.3 (7.4–10.3; n = 12)	9.8 (7.7–11.2; n = 18)
Day 13	12.7 (7.7–14.7; n = 4)	10.8 (7.3–13.2; n = 6)
Day 14	12.9 (8.6–17.1; n = 2)	15.1 (13.9–20.7; n = 7)

**Table 3:** Median (25–75% range) embryonic vesicle diameter in 80 pregnancies (excluding twin pregnancies) prior to embryo reduction at set time points on day 11 or 12. No significant difference existed in median embryo vesicle size between luteolytic and luteostatic mares (Kruskal–Wallis one-way ANOVA on Ranks; *p* > 0.05 all cases) at time periods throughout day 11 or 12, or overall for either day.

Day and Hour of Embryo Reduction		Median (25–75% Range; n) Embryo Diameter (mm) of Vesicles at the Time of Reduction in Mares That Became:	
		Luteolytic	Luteostatic
Day 11	0–4 h	5.7 (5.0–6.9; n = 9)	6.0 (4.4–7.3; n = 6)
	8–12 h	7.2 (6.1–7.7; n = 8)	5.8 (5.0–7.9; n = 8)
	16–20 h	7.7 (6.2–8.1; n = 4)	7.7 (5.9–9.1; n = 15)
	Overall	7.1 (5.5–7.7; n = 21)	6.8 (5.1–8.3; n = 29)
Day 12	0–4 h	8.6 (7.3–10.3; n = 3)	9.5 (5.5–11.1; n = 8)
	8–12 h	7.7 (6.7–8.5; n = 7)	9.9 (6.9–11.4; n = 5)
	16–20 h	10.9 (10.2–11.6; n = 2)	9.7 (7.6–11.9; n = 5)
	Overall	8.3 (7.4–10.3; n = 12)	9.8 (7.7–11.2; n = 18)

**Table 4:** Median (25–75% range) embryonic vesicle diameter and timing of reductions in three mares on specific days post-ovulation prior to embryo reduction. Only mares and timepoints in which ≥ four reductions resulted in luteolysis and ≥four resulted in stasis were analyzed. At each specific time point for an individual mare, no significant difference existed in the median embryo vesicle size between luteolytic and luteostatic mares (Kruskal–Wallis one-way ANOVA; *p* > 0.05 all cases). In one mare (PY), the reductions resulting in luteostasis occurred at significantly later timepoints on day 11 than those resulting in luteolysis (*p* = 0.029).

Mare ID	Day and Hours Post-Ovulation of Embryo Reduction	Median (25–75% Range; n) Embryonic Vesicle Diameter (mm) Prior to Reduction and Timing of Reduction (h Post-Ovulation) in Mares That Became:	
		Luteolytic	Luteostatic
AA	Day 12	7.2 (5.2–8.4; n = 4)	13.5 (8.7–11.9; n = 6)
	Hours	302 (294–304; n = 4)	300 (295–305; n = 6)
GY	Day 12	8.3 (7.5–9.8; n = 4)	9.6 (8.7–10.0; n = 6)
	Hours	304 (295–307; n = 4)	304 (299–309; n = 6)
PY	Day 11	4.8 (4.3–5.2; n = 5)	6.2 (4.7–7.1; n = 12)
	Hours	268 (268–272; n = 4) ^a^	276 (272–284; n = 6) ^b^

^a,b^ Different superscripts within a row indicate significant differences.

**Table 5:** Cycle length (ovulation [day 0] to the following ovulation) in seven individual mares following either embryo reduction not resulting in luteostasis or non-pregnant cycles. The mean ± the SEM values are reported. A two-way ANOVA showed mare ID but not cycle status-influenced cycle length (*p* < 0.01). A post-hoc Holm–Sidak pairwise comparison revealed which mares varied significantly (*p* < 0.05) in cycle length between each other.

Mare ID	Cycle Length (Mean Days ± SEM)		
	Pregnant, Embryo Reduction with No Luteostasis	Not Pregnant	All Cycles
ZA	20.7 ± 0.7 (n = 12)	20.8 ± 1.1 (n = 5)	20.8 ± 0.7 (n = 17) ^a^
CSO	21.3 ± 1.3 (n = 4)	20.8 ± 1.1 (n = 6)	21.0 ± 0.8 (n = 10) ^a^
PY	21.1 ± 1.3 (n = 4)	21.7 ± 1.8 (n = 2)	21.4 ± 1.1 (n = 6)
GY	21.4 ± 1.0 (n = 7)	21.9 ± 1.0 (n = 7)	21.6 ± 0.7 (n = 14) ^a^
RS	22.0 ± 1.5 (n = 3)	22.3 ± 1.3 (n = 4)	22.1 ± 1.0 (n = 7)
CO	24.0 ± 1.5 (n = 3)	21.3 ± 1.5 (n = 3)	22.7 ± 1.1 (n = 6)
AA	24.9 ± 0.9 (n = 8)	24.4 ± 0.8 (n = 10)	24.6 ± 0.6 (n = 18) ^b^
All mares	22.2 ± 0.5 (n = 41)	21.9 ± 0.5 (n = 37)	22.1 ± 0.3 (n = 78)

^a,b^ Different superscripts within a column indicate significant differences.

## Data Availability

The data presented in this study are available upon reasonable request from the corresponding author.
